# Supplementary material for: Heterogenic Origin of Micro RNAs in Atlantic Salmon (Salmo salar) Seminal Plasma
Source: Int J Mol Sci. 2020 Apr 15;21(8):2723. doi: 10.3390/ijms21082723 (PMC7216159; doi:10.3390/ijms21082723)
Supplement: Supplementary file 1 [file ijms-21-02723-s001.zip › Table S3.docx]

Table S1. Double DIG labelled LNA probe for in situ hybridization. Name, sequence, melting temperature, concentration of probe and hybridization temperature used for in situ experiment.

| Name | Sequence (5’ - 3’) | Concentration (nM) | Melting Temperature (^o^C) | Hybridization Temperature (^o^C) |
| --- | --- | --- | --- | --- |
| U6 snRNA | /5’DigN/CACGAATTTGCGTGTTCATCCTT/3’DigN/ | 10 | 84 | 55 |
| miR-15c-5p | /5’DigN/TCAAACCATGATGCGCTGCTA/3’DigN/ | 40 | 84 | 55 |
| miR-30d-5p | /5’DigN/AGCTTTCAGTCAAGGATGTTTACA/3’DigN/ | 40 | 84 | 55 |
| miR-92a-5p | /5’DigN/ACAGGCCGGGACAAGTGCAATA/3’DigN/ | 40 | 87 | 57 |
| miR-93a-5p | /5’DigN/CTACCTGCACAAACAGCACTTTT/3’DigN/ | 40 | 86 | 55 |
| miR-202-5p | /5’DigN/TTTTCCCATGCCCTATGCCTCT/3’DigN/ | 20 | 91 | 58 |
| miR-730-5p | /5’DigN/CACACAGCATGCACAATGAGGA/3’DigN/ | 40 | 84 | 55 |
