# Supplementary figures and images for: Heterogenic Origin of Micro RNAs in Atlantic Salmon (Salmo salar) Seminal Plasma
Source: Int J Mol Sci. 2020 Apr 15;21(8):2723. doi: 10.3390/ijms21082723 (PMC7216159; doi:10.3390/ijms21082723)

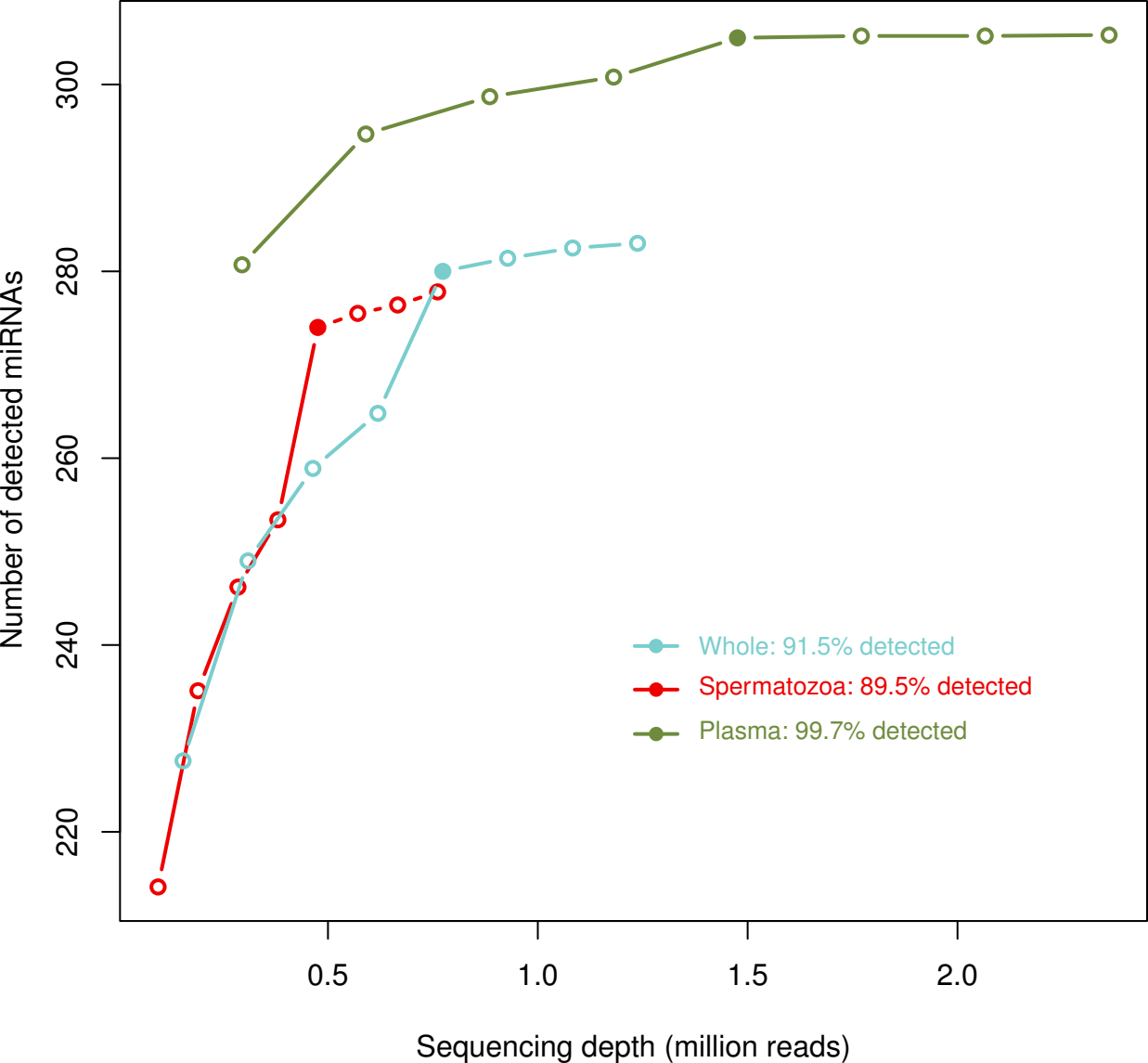

Supplement: Supplementary file 1 [file ijms-21-02723-s001.zip › Figure S1.pdf]

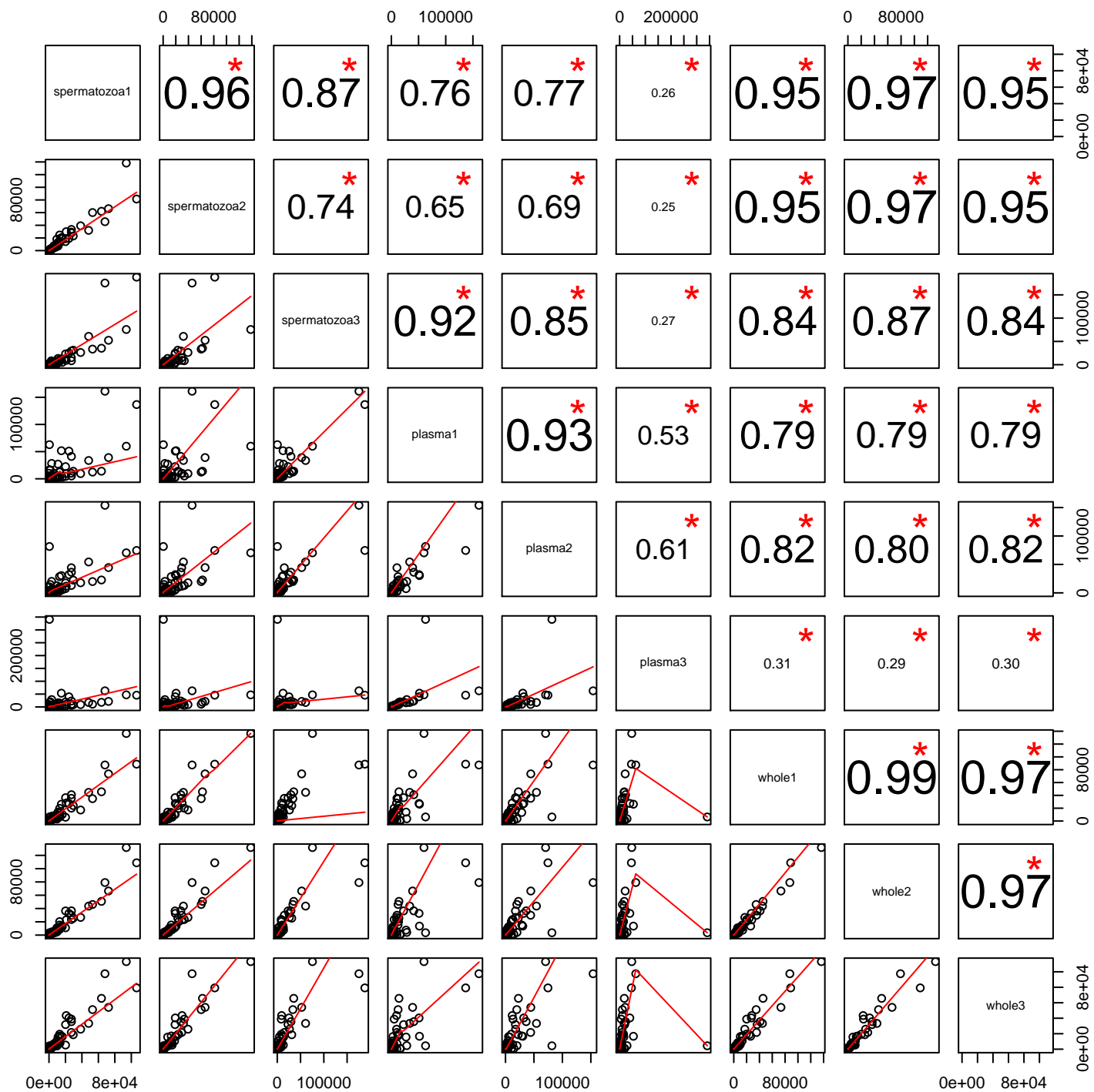

Supplement: Supplementary file 1 [file ijms-21-02723-s001.zip › Figure S2.pdf]

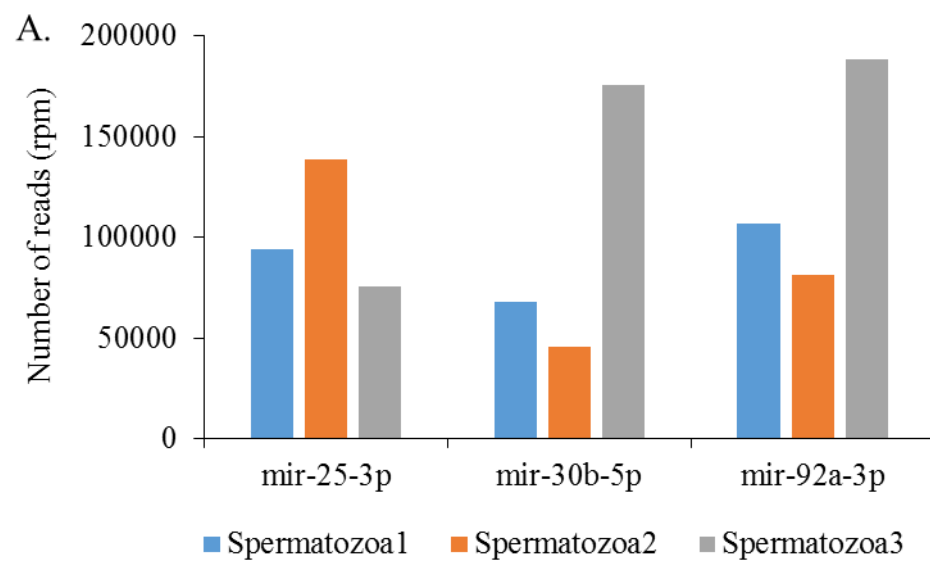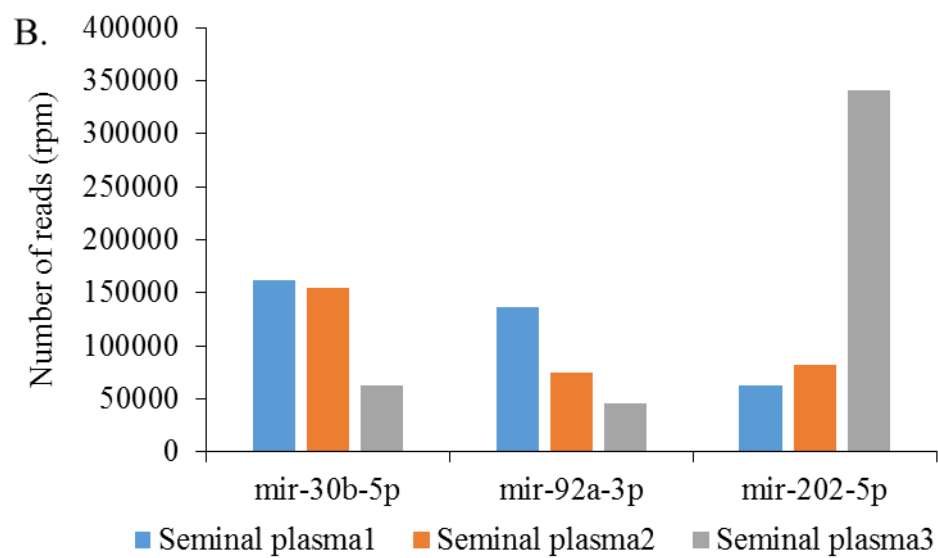

Supplement: Supplementary file 1 [file ijms-21-02723-s001.zip › Figure S3.pdf]

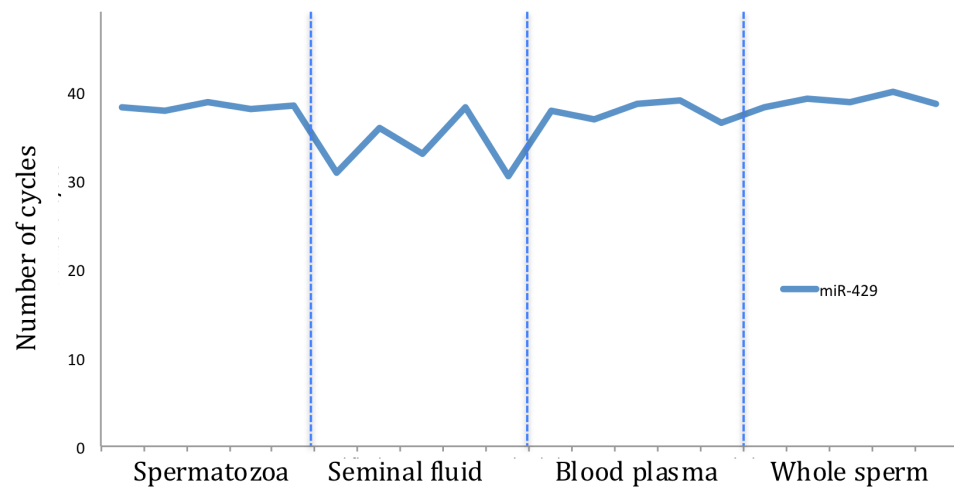

Supplement: Supplementary file 1 [file ijms-21-02723-s001.zip › Figure S6.pdf]

A. Juvenile Testis

B. Mature Testis

U6 snRNA

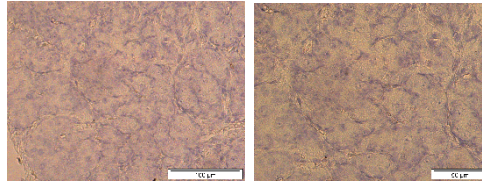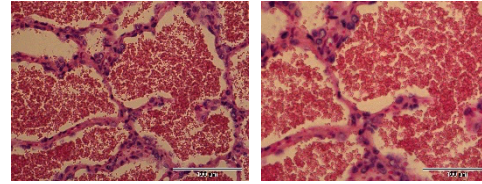

Scramble probe

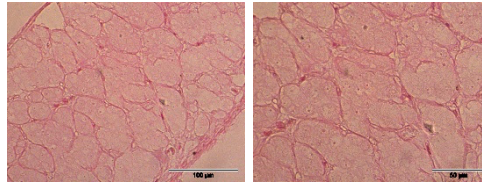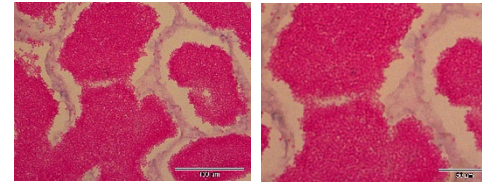

Supplement: Supplementary file 1 [file ijms-21-02723-s001.zip › Figure S4.pdf]

# Juvenile Ovary

A) ssa-miR-15c-5p

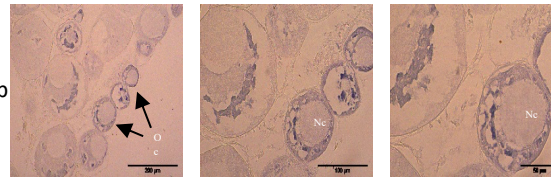

B) ssa-miR-30d-5p

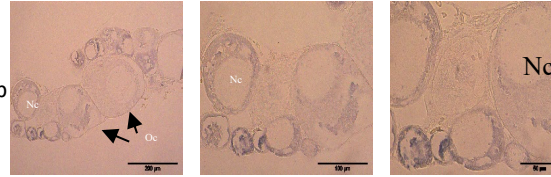

C) ssa-miR-92a-5p

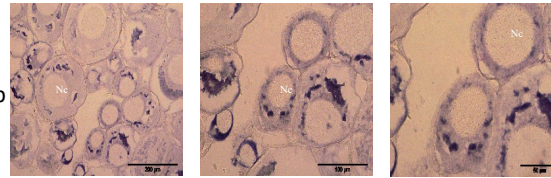

D) ssa-miR-93a-5p

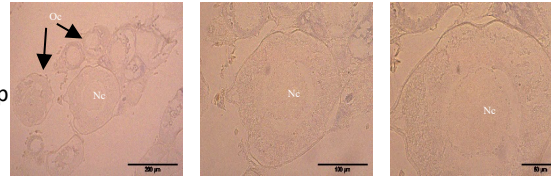

E) ssa-miR-202-5p

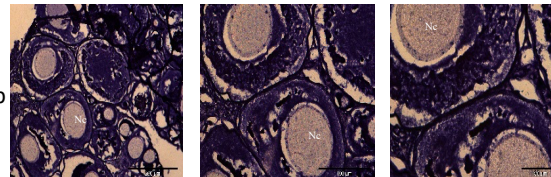

F) ssa-miR-730-5p

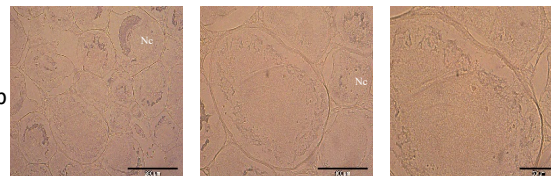

G) U6 snRNA

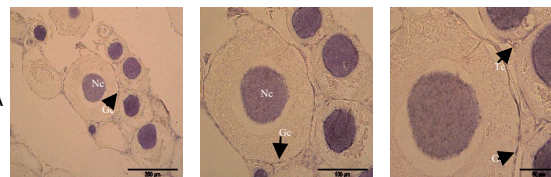

Supplement: Supplementary file 1 [file ijms-21-02723-s001.zip › Figure S5.pdf]
